# A Case of an Elderly Patient With Recurrent Spontaneous Coronary Artery Dissection

**DOI:** 10.7759/cureus.23633

**Published:** 2022-03-29

**Authors:** Hipólito R García Miranda, Pilar Valdovinos, Hernan Tajes, Josep M Alegret

**Affiliations:** 1 Cardiology, Hospital Universitario Sant Joan de Reus, Reus, ESP; 2 Family and Community Medicine, Hospital Lleuguer de Cambrils, Reus, ESP

**Keywords:** case report, elderly patients, fibromuscular dysplasia, spontaneous coronary artery dissection, recurrent spontaneous coronary artery dissection

## Abstract

Spontaneous coronary artery dissection (SCAD) is an underdiagnosed cause of myocardial infarction (MI), and its prevalence among women is increasing. Epidemiological information indicates that SCAD is responsible for one-third of MI cases in women of reproductive age. Little information is described on SCAD in elderly patients. The patient in the case presented here was an 81-year-old woman with a history of SCAD who presented with oppressive thoracic chest pain associated with electrical changes that derailed into ventricular fibrillation. Coronary angiography confirmed a SCAD recurrence, and conservative medical treatment was established. Different pathophysiological pathways have been proposed for SCAD extension or recurrence. Nonetheless, there is yet much to be discovered about this disease and its presentation in different age groups.

## Introduction

Spontaneous coronary artery dissection (SCAD) is defined as ¨the separation of layers of an epicardial coronary artery wall by intramural haemorrhage, with or without intimal tear, not associated with atherosclerosis, iatrogenic injury, or trauma, leading to acute coronary syndrome (ACS) [1]. The use of high-resolution imaging techniques, such as intraluminal optical coherence tomography (OCT), has provided support for the hypothesis that myocardial infarction (MI) is caused by an initial dissection or rupture in the vasa vasorum that results in a secondary intramural haemorrhage and the formation of an intramural haematoma, leading to lumen obstruction [2].

Epidemiological reports indicate that SCAD is responsible for less than 1% of all acute myocardial infarctions, but some cohort studies find SCAD to be responsible for one-fourth to one-third of MI cases with nonobstructive coronary artery disease in young women. At least 90% of patients are women between 47 and 53 years of age with a low prevalence of cardiovascular risk factors [1,3].

Evidence suggests a higher prevalence of SCAD in women with inflammatory diseases, such as systemic lupus erythematosus and sarcoidosis. In particular, fibromuscular dysplasia and connective tissue disease were taken into account among these patients. Recommendations based on expert consensus suggest screening the family members of patients with fibromuscular dysplasia and SCAD to detect signs or symptoms of SCAD. This case report was prepared following the CARE (CAse REport) guidelines [4].

## Case presentation

**Figure 1:**
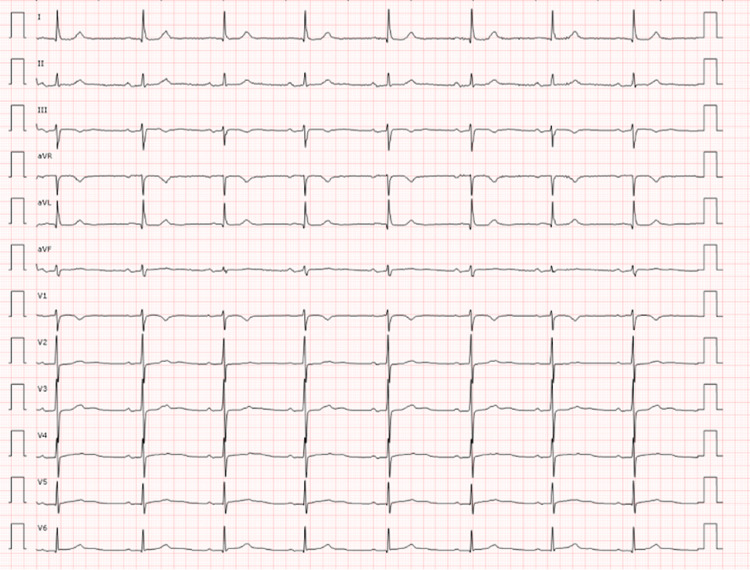
First electrocardiogram upon arrival

The patient was an 81-year-old woman with no known toxic habits who was overweight and had hypothyroidism, hyperuricaemia, osteoporosis, atrial fibrillation, and a long history of refractory arterial hypertension treated with nebivolol/hydrochlorothiazide 2/25 mg and olmesartan 40 mg daily. Three years prior to the current event, she presented with multiple self-limited, mild, oppressive thoracic pain episodes, without previous physical or stressor events, until one day the symptoms worsened, which was why emergency services were sought. Upon arrival, an electrocardiogram (EKG) was performed, and the results showed signs of acute lateral myocardial infarction (Figure 1).

**Figure 2:**
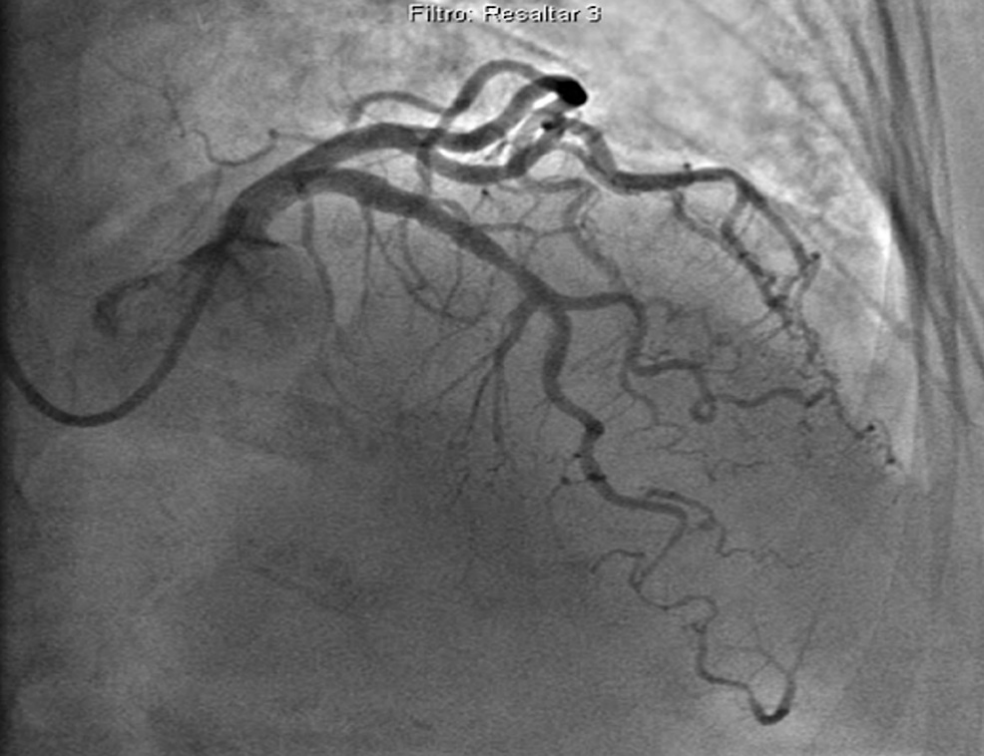
Coronary angiography of the first spontaneous coronary artery dissection showing a long stenosis of the mid-distal portion of the left anterior coronary artery

Double antiplatelet therapy (DAPT), nitroglycerin, heparin and morphine were administered, and the patient was urgently transported to the haemodynamic unit. Blood troponin I levels reached an initial peak of 1409 ng/ml, and urgent angiography was performed, showing one-vessel coronary artery disease of the mid-distal portions of the left anterior descending (LAD) coronary artery with 60% stenosis (Figure 2).

**Figure 3:**
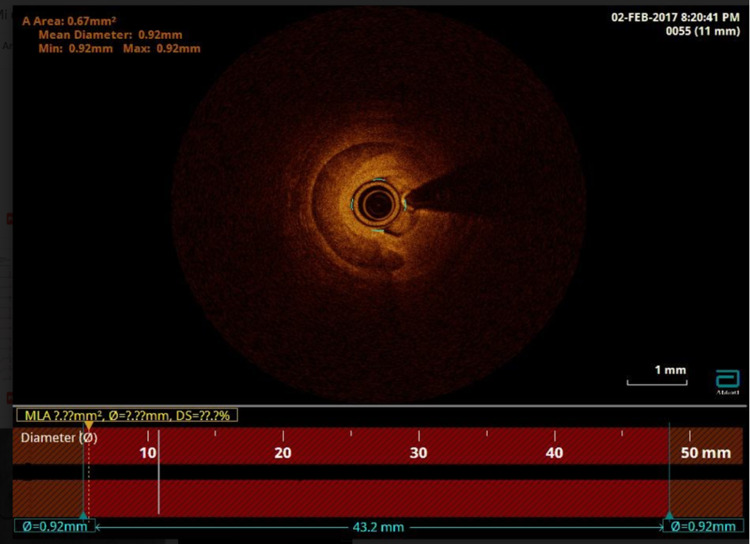
Intraluminal optical coherence tomography showing a hematoma of the left anterior coronary artery

Intraluminal OCT was performed in the distal LAD, finding a type 2B image of dissection throughout the stenosis and a suggestive image of distal exit (Figure 3). The OCT catheter was removed, and a control angiogram was performed, showing evident improvement in the diameter of the mid-distal LAD without conditioning a significant narrowing of the vessel after performing OCT and with normal distal flow; therefore, conservative medical treatment was chosen. Due to the correct clinical evolution associated with the normalization of electrical changes in EKG, hospital discharge was decided upon, along with progressive pharmacological treatment adjustment (nebivolol 5 mg, olmesartan/hydrochlorothiazide 40 mg/12.5 mg, doxazosin 8 mg daily). The patient did not follow a cardiac rehabilitation program after discharge.

**Figure 4:**
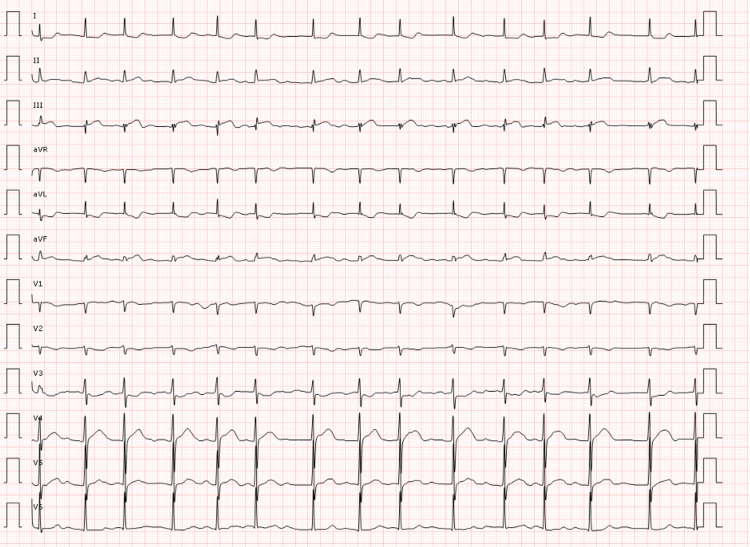
Electrocardiogram from the second event

Three years later, symptoms similar to previous events reappeared, and EKG was performed, showing atrial fibrillation and a 1-mm ST elevation in leads III and aVF; therefore, DAPT along with nitroglycerin was started (Figure 4). Upon arrival in the emergency room, the patient suffered from ventricular fibrillation that required advanced cardiopulmonary resuscitation and one defibrillation, after which rhythm was recovered, with atrial fibrillation and left bundle branch block. The blood troponin T level reached a peak of 833 ng/ml; on her way to the angiography centre, she presented a reversal to sinus rhythm with bradycardia and advanced atrioventricular block.

**Figure 5:**
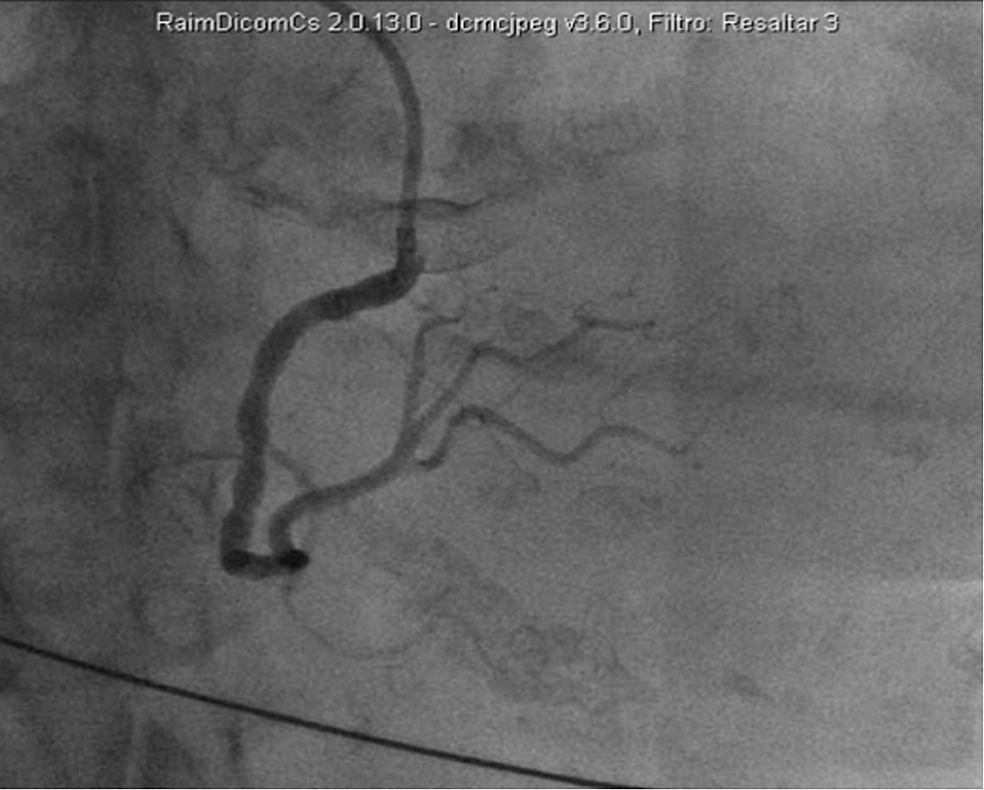
Coronary angiography of the second spontaneous coronary artery dissection showing a stenosis of the marginal branch of the right coronary artery

Angiography was performed, and a recurrent de novo SCAD event was found in the marginal branch of the right coronary artery (Figure 5). No treatment other than the implantation of a transient electrocatheter via the femoral route was performed, which was later removed due to the absence of symptomatic bradycardia. The echocardiogram showed severe dilation of the left atrium, a mild mitral insufficiency, hypokinesia limited to the inferobasal segment with a normal ejection fraction, and no pathological aortic images.

Given that this patient had a recurrent de novo spontaneous coronary dissection, extensive laboratory tests were performed, including tests of autoimmunity parameters, tumour markers, celiac disease, hyperaldosteronism, pheochromocytoma, and coagulation factors, all of which had negative results (except anti-TPO+, of 87, with normal thyroid function, which was explainable by known autoimmune hypothyroidism). Likewise, an angio-CT of the renal arteries was performed, which did not show findings suggestive of fibromuscular dysplasia or renal artery stenosis, and an abdominal CT was performed, which did not show adrenal masses. The clinical criteria for Marfan syndrome were not met. Thus, after the exclusion of various aetiologies, a diagnosis of recurrent de novo SCAD secondary to poorly controlled arterial hypertension was made. Finally, hospital discharge with continued outpatient management and cardiac rehabilitation was chosen.

## Discussion

To date, many cases of SCAD have been reported in the literature, taking into consideration three of the largest cohorts, including 511 women with SCAD thus far; 89.1% are women under 65 years of age and 10.9% are women over 65 years [5-8]. Therefore, keeping track of newly found SCAD cases in this population is important. Given that SCAD is an underdiagnosed disease that is becoming more prevalent, and now that we have better diagnostic tools, it is important that we consider every case reported throughout all age ranges [6]. Current evidence relates predisposing factors such as young age, physical and emotional stressors, and inflammatory and connective tissue diseases to SCAD [9]. Nonetheless, this case report suggests that none of the usual SCAD predisposing factors were involved, so we infer that poorly controlled arterial hypertension might have played a leading role throughout the SCAD events for this patient. Common cardiovascular risk factors and vascular frailty in elderly patients may play a role in the development of SCAD events, so correctly determining whether ACS in an elderly patient is caused by an SCAD event or if the cause is obstructive may be vital given that therapeutic options may differ among either option.

SCAD has been labelled as a disease of young females [10]. Although we currently might not consider SCAD as a cause of ACS in elderly individuals, the prevalence of the disease may increase if we take into account this age group and thoroughly assess angiography findings in elderly patients with ACS who did not have atherosclerotic disease findings.

Furthermore, there is a moderate risk (12%-27%) of recurrence among the reported cases in which patients have known predisposing factors, and this risk increases depending on the presence of coronary artery disease or arterial hypertension [11-13]. This is why studies focused on defining the pathophysiologic pathway of this disease as well as the optimal treatment to prevent recurrence are ongoing. Currently, beta blockers are the only pharmacological treatment regimen that has demonstrated a reduced risk of recurrence among these patients. The present case shows that even though correct pharmacological treatment was established after the first SCAD, a recurrent de novo SCAD event occurred; therefore, we hypothesize that focusing on cardiac rehabilitation and correct control of common cardiovascular risk factors may decrease the development and recurrence of these events.

## Conclusions

Spontaneous coronary artery dissection is a real and important cause to take into account in myocardial infarction in young female populations; nonetheless, the underevaluation of other age groups might play a role in underestimating the presence of this pathology, its presentation and its recurrence in the elderly population. Common cardiovascular risk factors control and cardiac rehabilitation may play a leading role in preventing SCAD de novo or recurrence in elderly patients. Further investigation remains to be performed to either confirm or deny these data.
